# Using asexual vertebrates to study genome evolution and animal physiology: Banded (*Fundulus diaphanus*) x Common Killifish (*F. heteroclitus*) hybrid lineages as a model system

**DOI:** 10.1111/eva.12975

**Published:** 2020-05-04

**Authors:** Anne C. Dalziel, Svetlana Tirbhowan, Hayley F. Drapeau, Claude Power, Lauren S. Jonah, Yayra A. Gbotsyo, Anne‐Marie Dion‐Côté

**Affiliations:** ^1^ Department of Biology Saint Mary's University Halifax NS Canada; ^2^ Département de biologie Université de Moncton Moncton NB Canada

**Keywords:** asexual reproduction, cytogenetics, epigenetics, gynogenesis, phenotypic plasticity

## Abstract

Wild, asexual, vertebrate hybrids have many characteristics that make them good model systems for studying how genomes evolve and epigenetic modifications influence animal physiology. In particular, the formation of asexual hybrid lineages is a form of reproductive incompatibility, but we know little about the genetic and genomic mechanisms by which this mode of reproductive isolation proceeds in animals. Asexual lineages also provide researchers with the ability to produce genetically identical individuals, enabling the study of autonomous epigenetic modifications without the confounds of genetic variation. Here, we briefly review the cellular and molecular mechanisms leading to asexual reproduction in vertebrates and the known genetic and epigenetic consequences of the loss of sex. We then specifically discuss what is known about asexual lineages of *Fundulus diaphanus x F. heteroclitus* to highlight gaps in our knowledge of the biology of these clones. Our preliminary studies of *F. diaphanus* and *F. heteroclitus* karyotypes from Porter's Lake (Nova Scotia, Canada) agree with data from other populations, suggesting a conserved interspecific chromosomal arrangement. In addition, genetic analyses suggest that: (a) the same major clonal lineage (Clone A) of *F. diaphanus x F. heteroclitus* has remained dominant over the past decade, (b) some minor clones have also persisted, (c) new clones may have recently formed, and iv) wild clones still mainly descend from *F. diaphanus* ♀ x *F. heteroclitu*s *♂* crosses (96% in 2017–2018). These data suggest that clone formation may be a relatively rare, but continuous process, and there are persistent environmental or genetic factors causing a bias in cross direction. We end by describing our current research on the genomic causes and consequences of a transition to asexuality and the potential physiological consequences of epigenetic variation.

## 1 GENERAL INTRODUCTION

Sexual reproduction, defined as “the union of two gametes and genomes” (Lehtonen, Jennions, & Kokko, 2012), is the predominant form of reproduction in eukaryotes (reviewed by Maynard‐Smith, 1978; Otto, 2009). This prevalence is surprising, because sexual reproduction has immediate fitness costs that can be avoided with asexual reproduction (reviewed by Lehtonen et al., 2012). Therefore, a major goal of evolutionary biology is to better understand why sex originally evolved and is maintained; this is often done by quantifying and comparing the costs and benefits of sexual versus asexual reproduction across different evolutionary time‐scales and environmental parameters using theoretical models (reviewed by Otto, 2009), laboratory‐based studies (reviewed by Sharp & Otto, 2016), and field studies (reviewed by Neiman, Meirmans, Schwander, & Meirmans, 2018). As environment‐specific factors are predicted to be important in the maintenance of sex, further comparisons of unisexual clonal lineages to their sexual congeners in the wild will be critical for evaluating hypotheses for why sex prevails in nature (Neiman, Lively, & Meirmans, 2017; Neiman et al., 2018). Beyond helping unravel the mystery of why so many eukaryotes reproduce sexually, natural clonal lineages have also been used to determine the genetic mechanisms by which sexual reproduction breaks down and asexual fertile eggs are produced (reviewed by Avise, 2012; Dawley & Bogart, 1989; Neaves & Baumann, 2011; Neiman, Sharbel, & Schwander, 2014). Furthermore, asexual clones can be used to address other biological questions that require genetically identical replicate individuals. In such studies, natural clonal vertebrates may be preferred over inbred/isogenic genetically identical, laboratory‐bred lines because they have evolved in the wild, so represent real evolutionary outcomes, unlike laboratory strains that might have low fitness in natural environments (reviewed by Laskowski, Doran, Bierbach, Krause, & Wolf, 2019).

The goal of this paper is not to discuss the evolutionary paradox of sex, but to highlight how studying naturally occurring, asexual vertebrate lineages can provide insight into the mechanisms by which genomes evolve and epigenetic modifications influence vertebrate physiology. In the first section of this paper, we review general trends in asexually reproducing vertebrates, including the cellular and molecular mechanisms leading to asexual reproduction, and the known genetic and epigenetic consequences of the loss of sex. We then review the benefits of using asexual fishes as model organisms and discuss what is known about *Fundulus diaphanus x F. heteroclitus* clonal lineages, including new data from our laboratories. We end by outlining our current research using these asexual fish to study the genomic causes and consequences of a transition to asexuality and the effects of epigenetic variation on phenotype. For readers interested in the broader topic of asexuality in multicellular organisms, including the causes and consequences of this mode of reproduction in invertebrates and plants, we suggest reviews by Neiman et al. (2014), Neiman et al. (2017), Schwander, Marais, and Roze (2014), and Schmidt, Schmid, and Grossniklaus (2015).

## 2 INTRODUCTION TO ASEXUAL REPRODUCTION IN VERTEBRATES

The majority of vertebrates reproduce sexually, but there are about 100 known asexual lineages of fish, reptiles, and amphibians (Avise, 2008, 2015; Dawley & Bogart, 1989; Neaves & Baumann, 2011; Vrijenhoek, Dawley, Cole, & Bogart, 1989). The lack of avian or mammalian unisexual clonal lineages is hypothesized to be the result of developmental and genetic constraints in these endothermic lineages, such as the developmental lethality associated with errors in mammalian genomic imprinting (Kono et al., 2004) and the bird sex determination system (female heterogamety may prevent successful asexual reproduction; see Engelstädter, 2008).

Asexual lineages of vertebrates are, to the best of our knowledge, almost exclusively the result of interspecific hybridization between parental species that tend not to be sister‐taxa (reviewed by Avise, 2015; Laskowski et al., 2019; Neaves & Baumann, 2011). This observation, in combination with data showing that the proportion of unreduced gametes increases and hybrid fecundity decreases with parental species divergence, led Moritz et al. (1989) to suggest the “balance hypothesis.” This hypothesis predicts that asexual clone formation is a form of reproductive isolation that occurs when reproductive incompatibilities have accumulated to the extent where normal gametogenesis is disrupted, but hybrids are still viable and asexually fertile (Vrijenhoek, 1989). In particular, divergence between parental species may interfere with homologous chromosome alignment, homology search and subsequent crossovers during meiosis in F1 hybrids (Dion‐Côté & Barbash, 2017), which may increase the probability of a transition to asexuality by destabilizing meiosis (Janko et al., 2018). The ability to form these asexual lineages is also hypothesized to be phylogenetically constrained, such that only certain taxa have the genetic and developmental machinery required to form fertile clones (reviewed by Engelstädter, 2008). The specific genetic and cellular mechanisms leading to the formation of most unisexual vertebrates are still under study (see Section 2.1 “Cellular and molecular mechanisms of asexual reproduction in vertebrates”), but evidence that hybridization is critical for the initiation of clonality in vertebrates continues to mount (Janko et al., 2018; Neiman et al., 2014).

### 2.1 Cellular and molecular mechanisms of asexual reproduction in vertebrates

**FIGURE 1:**
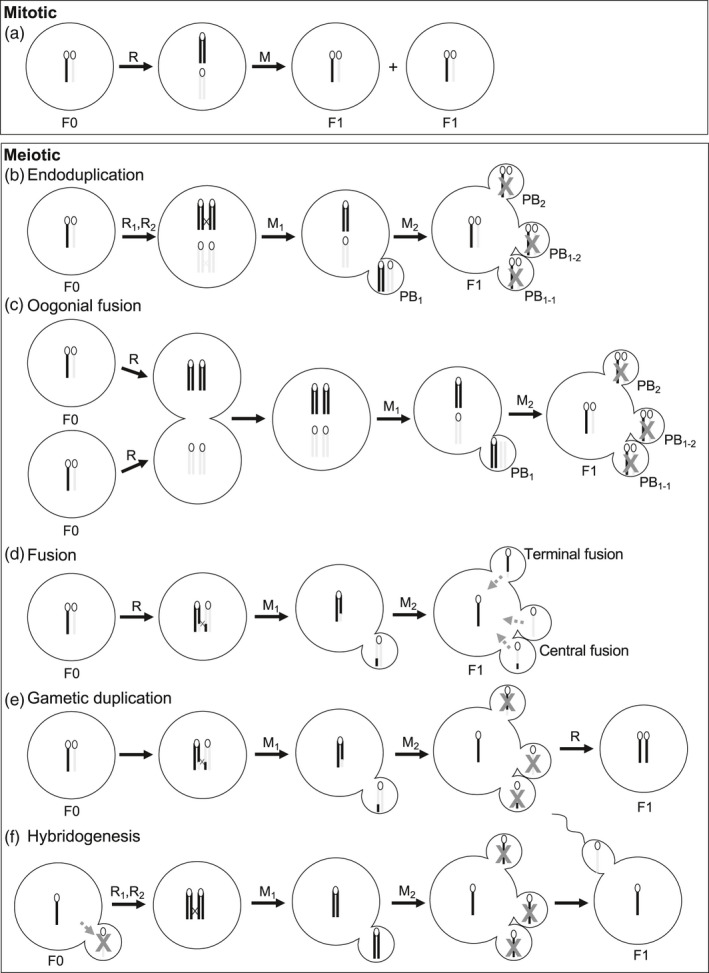
Modes of asexual egg production. This figure is inspired and adapted from Neaves & Baumann, (2011) and Neiman et al., (2014). Different shades indicate homologous chromosomes. F0: parental generation, F1: progeny , R: DNA replication, M: mitosis (upper panel) and meiosis (lower panel). PB: polar body. Upper panel: (a) Under mitotic egg production, the cell divides according to regular mitosis, and both ploidy and heterozygosity are maintained. Progeny are perfect clones of their mother. Lower panel: Asexual egg production by a modified version of meiosis may yield to variable results. (b) Under endoduplication, the germ cell undergoes an additional round of DNA replication prior to entering meiosis (R_1_, R_2_). Duplicate chromosomes recombine together and therefore heterozygosity is maintained and the progeny are perfect clones. (c) Under oogonial fusion (which has not been observed in vertebrates in nature), premeiotic oogonia fuse, and sister chromosomes recombine as in endoduplication, also resulting in perfect clonality and the maintainenance of heterozygosity. (d) Under fusion, the early meiotic program is maintained (including meiotic recombination between homologous chromosomes) and the maturing egg fuses with either of the first polar bodies (central fusion) or the secondary polar body (terminal fusion). Progeny heterozygosity and genetic makeup will vary depending on the exact product that fuses with the oocyte. (e) Under gametic duplication, haploid gametes are produced following meiosis, and their genome is subsequently duplicated. Genetic makeup may vary (depending on whether a recombinant sister chromatid ends up into the egg) but heterozygosity is completely lost. (f) Finally, hybridogenesis is a particular case in which the paternal genome is eliminated prior entry into meiosis. The resulting progeny are thus perfect half clones (no recombination can occur) but differ in their paternal complement

**FIGURE 2:**
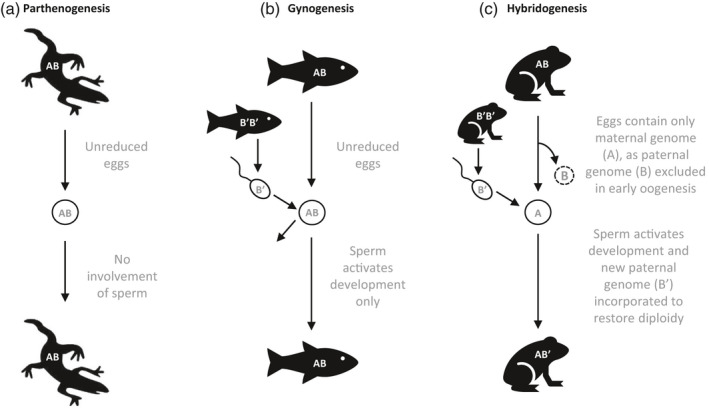
Modes of development initiation in asexual vertebrates. Eggs may (a) develop without any contribution from sperm during parthenogenesis, (b) have sperm initiate development during gynogenesis, or (c) develop after sperm activation and paternal genetic complement inclusion during hybridogenesis, such that only the maternal genome is clonally transmitted. Modeled after Avise, (2008), Laskowski et al., (2019), and Neaves & Baumann, (2011)

Asexuality has evolved independently many times in vertebrates (Avise, 2015). Consequently, the mechanisms underlying the modes of egg production and activation of embryonic development vary across—and sometimes even within—lineages. Here, we discuss the most common forms of egg production (Figure 1) and development initiation (Figure 2) in asexual vertebrates, while considering ploidy maintenance and the genetic impacts of different modes of asexuality.

#### 2.1.1 Modes of asexual egg production

If eggs are produced mitotically, ploidy and genetic makeup are readily maintained. However, mitotic egg production is not known to occur in asexual vertebrates (Neiman et al., 2014). Thus, asexual vertebrate eggs likely all undergo some modified form of meiosis. Meiosis is a reductional cell division, so a frequent prerequisite to asexual reproduction in vertebrates is the production of unreduced eggs (i.e., same ploidy as the mother—either diploid or polyploid). These eggs may, or may not, be genetically identical to their mother, depending on whether recombination has occurred between sister (see next paragraph) or homologous chromosomes, and the specific mechanism used to maintain ploidy (see Engelstädter, 2008; Neaves & Baumann, 2011; Neiman et al., 2014 for more detailed explanations and diagrams).

Under endoduplication (also termed endoreplication), which appears to be the most frequent mechanism used by asexually reproducing vertebrates to maintain ploidy, germ cells undergo an additional round of DNA replication prior to entering meiosis (Neaves & Baumann, 2011). This means that the cell entering meiosis has twice the amount of DNA there would be in a sexually reproducing organisms (Figure 1); this process has been reported in a number of vertebrates, including fishes in the family *Cobitidae* (Arai & Fujimoto, 2013; Dedukh et al., 2020; Itono et al., 2006; Juchno, Arai, Boron, & Kujawa, 2017) and the genus *Poeciliopsis* (Cimino, 1972b). After endoduplication, meiotic recombination occurs between duplicate chromosomes (“sister chromosomes”), instead of homologous chromosomes (Figure 1b). Sister chromosomes are predicted to align on the metaphase plate in meiosis I (Kuroda, Fujimoto, Murakami, Yamaha, & Arai, 2018), resulting in an egg that is an exact genetic and chromosomal copy of its mother. Other processes besides endoduplication may also yield unreduced eggs, but these have been poorly characterized in vertebrates at present. For example, endoduplication could be substituted by oogonial fusion (oogonia are premeiotic germ cells), a process that would be genetically equivalent, but has not been reported in vertebrates (Figure 1c; Neaves & Baumann, 2011). As well, Newton et al. (2016) found that cells entering meiosis in parthenogenetic whiptail lizards were tetraploid, similarly to a closely related sexual species, meaning that the additional round of DNA replication may be a stochastic event that occurs during, and not before, meiosis. Note that under hybridogenesis, a form of “semi‐asexual reproduction” (see Section 2.1.2 “Development initiation in asexual vertebrates”), reduced eggs are produced by premeiotic exclusion, a mechanism by which the paternal chromosome complement is excluded, yielding reduced, nonrecombinant eggs (Figure 1f). These eggs are subsequently fertilized, and the paternal genome incorporated, to generate a diploid embryo, such as in some *Poeciliopsis* and *Squalius* lineages (Alves, Coelho, & Collares‐Pereira, 2001; Cimino, 1972a; Schultz, 1977; reviewed in Lamatsch & Stöck, 2009).

There are other potential mechanisms that may produce eggs with a ploidy similar to the mother's somatic cells, which have only been reported in invertebrates to date (Figure 1d,e; central fusion, terminal fusion and gamete duplication; reviewed by Neaves & Baumann, 2011; Neiman et al., 2014; Suomalainen, Saura, & Lokki, 1987). To understand other types of ploidy restoration and their genetic consequences, it is important to remember that (a) female meiosis is characterized by the production of a single egg, and degeneration of the other three products (i.e., polar bodies), and (b) meiotic recombination normally occurs between homologous chromosomes (not sister chromosomes), such that recombinant chromosomes bear new allele combinations. Under central fusion, the egg fuses with a polar body bearing non‐sister chromatids, so the resulting embryo may, or may not, be genetically identical to their mother depending on whether recombinant chromatids end up in the egg. Under terminal fusion, meiotic products bearing sister chromatids postrecombination fuse, yielding an embryo that is genetically different from their mother (resulting in a substantial decrease of heterozygosity). For both terminal and central fusion, fusion between two polar bodies to give rise to the embryo is also a possibility but has not been reported in vertebrates (Stenberg & Saura, 2009). Finally, under gamete duplication (Figure 1e), there is no fusion of meiotic products, and ploidy is instead restored by duplication of the egg's genetic material, giving rise to an entirely homozygous embryo.

#### 2.1.2 Development initiation in asexual vertebrates

Among vertebrates, the mode of development initiation during asexual reproduction comes in two major flavors (Figure 2): (a) parthenogenesis (spontaneous development initiation), and (b) gynogenesis (requires sperm activation of development). There are also two systems that integrate elements of both asexual and sexual reproduction: hybridogenesis and kleptogenesis (Avise, 2008). Hybridogenesis requires sperm activation and incorporates the paternal genetic complement, without recombination with the maternal genome, for just one generation (see Figure 2). For this reason, hybridogenesis is often referred to as hemiclonal reproduction (Avise, 2008). Kleptogenesis, a particular reproductive mode where unisexual females appear to sporadically “steal” male genomic DNA from congener species, is found in some salamanders in the genus *Ambystoma* (Bogart, Bi, Fu, Noble, & Niedzwiecki, 2007). Since kleptogenesis appears to be restricted to these salamanders, we will not discuss it further in this review focused upon fishes (see Avise, 2008 for further information).

Parthenogenesis is an umbrella term describing the development of an individual from an unfertilized egg (Maynard‐Smith, 1978). As far as we know, parthenogenesis in vertebrates is restricted to the production of females without the contribution of a male (i.e., thelytoky; Avise, 2015). All known parthenogenetic vertebrates are squamates, mainly lizards (Avise, 2008). If eggs were to be produced mitotically (i.e., apomixis), there would be no ploidy reduction and the resulting progeny would be genetically identical to their mother. As eggs from asexual vertebrates are produced meiotically (i.e., automixis), the genetic makeup of the progeny may, or may not, be identical to their mother, depending on the exact cellular and molecular mechanisms involved in egg production (see previous section). For example, in *Aspidoscelis* whiptail lizards, ploidy and heterozygosity are respectively maintained by first undergoing two premeiotic replication cycles (i.e., premeiotic endoduplication) followed by the strict pairing of sister chromosomes, and recombination among sister chromosomes (Lutes, Neaves, Baumann, Wiegraebe, & Baumann, 2010). This sister chromosome pairing maintains elevated heterozygosity and linkage disequilibrium, as opposed to canonical meiosis where homologous chromosomes pair and recombine (Lutes et al., 2010).

Gynogenesis is similar to parthenogenesis with the key difference being that females produce unreduced eggs that still require sperm, which leads to the activation of Ca^2+^ signaling pathways initiating embryo development (Stricker, 1999). Sperm may also contribute a centrosome, an organelle essential for chromosome segregation (Engelstädter, 2008; Lamatsch & Stöck, 2009). Sperm normally comes from a closely related sexually reproducing species, but the paternal complement is not incorporated in the developing embryo; ploidy is therefore maintained (Avise, 2008). This mode of unisexual reproduction has been reported in more than half of asexually reproducing vertebrates, including several fish and amphibian lineages (Avise, 2015). For example, some female *Chrosomus eos‐neogaeus* hybrids (formerly known as *Phoxinus*) produce unreduced eggs that must be activated by *C. eos* sperm (Dawley, Jack Schultz, & Goddard, 1987). However, gynogenesis is not always maintained in *Chrosomus* hybrids, as in some cases the paternal genetic complement is incorporated, yielding triploid individuals (reviewed by Lamatsch & Stöck, 2009). In other cases, haploid eggs are produced by triploid female *Chrosomus eos‐neogaeus* hybrids and fertilized by *C. eos* sperm, yielding diploid fish with fully *eos* nuclear genomes and *neogaeus* mitochondrial genomes (i.e., cybrids, Goddard, Megwinoff, Wessner, & Giaimo, 1998). Engaging in the risky aspects of sex, without any of the proposed benefits, makes the persistence of gynogenesis especially interesting from an ecological and evolutionary perspective (Schlupp, 2005). There is also evidence that at least one vertebrate, the cyprinid *Squalius alburnoides,* may reproduce by androgenesis (Morgado‐Santos, Carona, Vincente, & Collares‐Pereira, 2017); this form of reproduction results in offspring with only male nuclear, and sometimes mitochondrial chromosomes, so is the “flip‐side of gynogenesis” (reviewed by Lehtonen, Schmidt, Heubel, & Kokko, 2013).

Females that reproduce by hybridogenesis produce reduced eggs carrying the maternal chromosome complement that has not undergone recombination; the paternal complement is eliminated from the egg before meiosis is initiated (Lafond, Hénault, Leung, & Angers, 2019; Lavanchy & Schwander, 2019). Eggs are fertilized by sperm, and the new paternal complement is subsequently incorporated in the diploid organism but ejected from the germ line. Thus, hybridogens are effectively hemi‐clones, as only the maternal genome is transmitted to the progeny, but not entirely asexual organisms, strictly speaking, as adults have genomes from two parents. As with gynogens, sperm is needed to release arrest of meiosis II and activate embryonic development, and might also contribute a centrosome (Lamatsch & Stöck, 2009). Hybridogenesis is well documented in fish of the genera *Poeciliopsis*, in which the formation of a unipolar spindle precluding meiosis leads to the transmission of strictly the maternal complement to the oocyte, a mechanism referred to as premeiotic exclusion (Cimino, 1972a). Therefore, meiosis initiates with a single chromosome complement, and cell division in meiosis I is suppressed (Cimino, 1972a). The oocyte is haploid, carrying the maternal complement, and diploidy is restored upon fertilization by incorporating the new father's genetic material (Cimino, 1972a).

### 2.2 The genomic consequences of asexuality

#### 2.2.1 Ploidy

Whether polyploidy is a cause or consequence of asexual reproduction has been widely debated (reviewed by Avise, 2009). However, multiple lines of evidence, including cytonuclear data and the presence of asexual taxa with the same ploidy level as sexual parents, suggest that polyploidy is normally a consequence of hybrid‐induced asexuality, especially in vertebrates (reviewed by Avise, 2008; Neiman et al., 2014). Despite this evidence, it remains extremely difficult to disentangle the cause(s) from the consequence(s) of asexuality, hybridization and polyploidization (e.g., Lundmark & Saura, 2006). If polyploidy is a consequence of asexual reproduction, the prevalence of polyploids among asexual organisms may suggest polyploidy is advantageous (e.g., by increasing allelic diversity at immune genes, e.g., King, Seppälä, & Neiman, 2012) or simply better tolerated in asexual organisms (e.g., because of reduced constraints with respect to sex determination; Stenberg & Saura, 2009).

Asexual vertebrate polyploids are generally thought to have evolved via a “primary‐hybrid route”, in which diploid, hybrid mothers first produce unreduced eggs, and then additional events led to polyploid lineages (Schultz, 1969; reviewed in Avise, 2008, 2015; Lamatsch & Stöck, 2009). Polyploids (typically triploids) often occur in sperm‐dependent asexual lineages (but exceptions exist, e.g., in lizards of the genus *Aspidoscelis*), where eggs are frequently exposed to sperm. There are two general mechanisms by which “primary hybrid” polyploid asexual lineages may form: genome addition and genome duplication (reviewed in Avise, 2008). Under the “genome‐addition hypothesis,” unreduced eggs (AB) are produced and paternal genome is subsequently added (A' or B'), yielding a triploid embryo (ABA' or ABB'). Under the “genome‐duplication hypothesis,” an equational meiotic division (i.e., meiosis II, so sister chromatids do not segregate) is suppressed, yielding unreduced, highly homozygous eggs (AA or BB) which are subsequently fertilized (by A' or B') to give rise to a triploid embryo (AAB' or A'BB; note AAA' and BBB' could theoretically be formed but are not found in nature; Avise, 2008). A major difference between genome addition and genome duplication is that the heterozygosity of the homospecific chromosome set will be similar to the parental species in the former and close to null in the latter (except for de‐novo mutations).

#### 2.2.2 Elevated heterozygosity

As almost all known vertebrate asexual lineages are hybrids, they have elevated heterozygosity compared to parental species (note that this is assuming orthologous sequences are present in both parental species; if not, individuals would have elevated hemizygosity). What is perhaps more surprising is that this heterozygosity is generally retained (Warren et al., 2018), suggesting that, in asexual vertebrates, meiosis is usually modified in ways that maintain heterozygosity. While mitotic parthenogenesis, endoduplication and oogonial fusion maintain heterozygosity, central and terminal fusion would lead to a progressive loss of heterozygosity, and gamete duplication would result in an immediate loss of heterozygosity. As mitotic parthenogenesis has never been reported in vertebrates, egg production in asexually reproducing vertebrates is thought to proceed mostly by either premeiotic endoduplication or oogonial fusion (Neaves & Baumann, 2011). Altogether, these observations suggest that elevated heterozygosity may be advantageous in asexual vertebrates, perhaps by buffering deleterious recessive mutations.

#### 2.2.3 Deleterious mutation accumulation

Under most types of asexual reproduction, meiotic recombination among homologous chromosomes is disrupted, so linkage disequilibrium is complete. Thus, new allele combinations are not tested and deleterious alleles can no longer be purged from a lineage (Felsenstein, 1974; Hill & Robertson, 1966). As a result, deleterious alleles tend to accumulate, a phenomenon termed Muller's ratchet (Keightley & Otto, 2006; Muller, 1964). Mutations are also predicted to accumulate due to the reduced effective population size of asexual lineages (Balloux, Lehmann, & Meeus, 2003; Orive, 1993). The adaptive potential of asexual lineages is thus thought to be limited, leading to the classic prediction that asexual lineages should be short‐lived, evolutionary dead ends (Maynard‐Smith, 1978). Genetic, and more recently, genomic studies, have revealed that asexual lineages do tend to accumulate more deleterious mutations (Bast et al., 2018; Hartfield, 2016). Alternatively, allelic gene conversion (i.e., mitotic recombination, the process by which a sequence replaces the homologous sequence) can accelerate the spread of beneficial mutations in asexual lineages (Mandegar & Otto, 2007) and counteract the accumulation of deleterious mutations, a phenomenon that has been observed in ancient bdelloid rotifers (Flot et al., 2013). Similarly, a recent genomic study on the gynogenetic Amazon molly (*Poecilia formosa*) revealed a global lack of degeneration in this rather ancient asexual lineage (~500,000 generations, Warren et al., 2018). One explanation could be that paternal introgression (“stealing” of DNA fragments from the sexually reproducing male fertilizer, similarly to kleptogenetic salamanders) is more frequent than expected, thus mitigating deleterious mutation accumulation (Warren et al., 2018). Overall, while we do have solid theoretical foundations predicting deleterious mutation accumulation in asexual lineages, empirical data remains equivocal. More studies are clearly needed to understand the persistence of asexual lineages despite their predicted genomic decay.

#### 2.2.4 Transposable element load

Transposable elements (TEs) are selfish genetic elements that can multiply within a genome and colonize new genomes, thus increasing in frequency in a population, without providing any benefit to their host (Doolittle & Sapienza, 1980; Orgel & Crick, 1980). These properties lead to the hypothesis that asexual lineages should have a reduced TE load (Hickey, 1982), as, in the absence of sex, TEs can no longer colonize new genomes, and their evolutionary fate is then tightly linked to the survival of their host lineage. This prediction has received support in yeast, where Bast, Jaron, Schuseil, Roze, and Schwander (2019) found that asexual reproduction appears to select for an increased excision rate of TEs, leading to a reduced TE load over time. In asexually reproducing vertebrates, two observations complicate this prediction: (a) TEs tend to accumulate in nonrecombining regions of the genome (Bachtrog, 2003; Charlesworth, Jarne, & Assimacopoulos, 1994; Charlesworth, Lapid, & Canada, 1992), and (b) TE de‐repression (i.e., reactivation or mobilization) has repeatedly been observed in interspecific crosses (Arkhipova & Rodriguez, 2013; Dennenmoser et al., 2017; Dion‐Côté, Renaut, Normandeau, & Bernatchez, 2014; Kelleher, Edelman, & Barbash, 2012; O'Neill, O'Neill, & Graves, 1998), and all known asexual vertebrate lineages are hybrids. It is worth noting that such TE de‐repression may temporarily increase mutation rate, so could potentially contribute to asexual hybrid lineages adaptation (Rey, Danchin, Mirouze, Loot, & Blanchet, 2016; Stapley, Santure, & Dennis, 2015). It therefore remains unclear what the net balance of TE content is in asexual (hybrid) vertebrates compared to their parental species.

#### 2.2.5 Genome architecture

If meiosis is lost, then global genome organization (e.g., the karyotype and gene synteny) and other structures important for meiosis (e.g., meiotic proteins) may degenerate, as a result of reduced constraints and relaxed selection, respectively (Judson & Normark, 1996). In particular, ectopic recombination should be less deleterious in asexual lineages in which constraints on homologous chromosome alignment during meiosis are gone (due to sister chromosome alignments instead of homologous chromosome alignment during meiosis 1). One might then predict asexual lineages to have highly rearranged genomes. Indeed, this has been observed in asexual nematodes closely related to *Caenorhabditis elegans* that all have a single chromosomes (compared to 5–7 chromosomes in related sexually reproducing species; Fradin et al., 2017). The genome of one of these species has been sequenced, revealing a highly rearranged single chromosome with high level of heterozygosity (note that the two alleles are now on the same chromosome) that arose by the fusion of the six ancestral chromosomes (Fradin et al., 2017). A similarly highly rearranged genome, incompatible with conventional meiosis, has also been documented in ancient bdelloid rotifers (Flot et al., 2013), and high rates of genome rearrangement have also been reported in parthenogenetic aphids (Blackman, Spence, & Normark, 2000). Yet, Majtánová et al. (2016) reported no increased rate of chromosomal change in asexual *Cobitis* fish. The question of whether asexual reproduction yields to an increased rate of genome rearrangements in vertebrates therefore remains open.

### 2.3 The effect of asexual reproduction on vertebrate epigenetic variation

Epigenetic modifications, or “non‐genetic influences” (Burggren & Crews, 2014), can be defined as “gene regulation determinants that can be transmitted through mitosis and meiosis, such as covalent chemical modifications to the DNA, histone posttranslational modifications (PTMs) and diverse RNA species” (e.g., small RNAs, including piRNAs and miRNAS; Best et al., 2018; Lind & Spagopoulou, 2018; Skvortsova, Iovino, & Bogdanović, 2018; Verhoeven, Vonholdt, & Sork, 2016). These epigenetic marks do not involve changes in DNA sequence and may influence phenotype by suppressing or facilitating the expression of associated genes (Feil & Fraga, 2012). Epigenetic variation spans a spectrum from being fully genetically controlled to being fully autonomous from DNA sequence variation, and in this review we focus only on fully DNA‐dependent and fully autonomous epigenetic mechanisms for simplicity (see Richards, 2006 for a further discussion). From an evolutionary standpoint, DNA‐dependent epigenetic variation acts as all other genetically based changes (Richards, 2006), and may result from the mechanisms discussed in the previous section (see Section 2.2. “The genomic consequences of asexuality”). However, autonomous epigenetic modifications may further explain phenotypic variability, and potentially heritability, beyond what can be understood by studying genetics alone, so better characterizing the causes and consequences of this type of epigenetic change has become a current focus in evolutionary biology (Verhoeven et al., 2016).

Prior to discussing the potential effects of asexuality on autonomous epigenetic variation, it is important to describe the types of non‐DNA‐dependent epigenetic modifications that may occur. Autonomous epigenetic modifications can be classified as randomly occurring (“untargeted”; Shea, Pen, & Uller, 2011) or induced by the environment in a targeted manner (termed “environment‐directed”; Feinberg & Irizarry, 2010; Shea et al., 2011), such that specific environmental exposures lead to characteristic epigenetic modifications (reviewed by Feil & Fraga, 2012). In both cases, these epimutations may lead to beneficial, neutral or negative effect on organismal fitness (reviewed by Duncan, Gluckman, & Dearden, 2014; Verhoeven & Preite, 2014). Targeted epimutations are predicted to more commonly lead to beneficial phenotypic plasticity (Beldade, Mateus, & Keller, 2011; Ghalambor, McKay, & Carroll, 2007; Gibert, Mouchel‐Vielh, De Castro, & Peronnet, 2016), under the assumption that animals have evolved to sense and respond appropriately to environmental variability (Feil & Fraga, 2012). However, targeted epimutations can also have a negative effect on fitness, as evidenced by the environment‐induced epigenetic changes leading to human diseases (Cavalli & Heard, 2019). On the other hand, higher rates of untargeted or stochastic epimutations are predicted to be similar to random mutations and be associated with bet‐hedging; stochastic epigenetic modifications may be induced by stressful environments or unpredictable environments (Vogt, 2017).

Epigenetic changes can also be classified based upon their persistence. Some epigenetic modifications may persist through mitosis (intragenerational inheritance), through meiosis to the subsequent generation (intergenerational inheritance or meiotic epigenetic inheritance), and even through meiosis to future generations that have never experienced the stimulus leading to the initial epigenetic change (transgenerational epigenetic inheritance; Best et al., 2018; Skvortsova et al., 2018). However, the likelihood of transmission decreases from intra‐ to inter‐ to transgenerational inheritance, as substantial epigenetic resetting occurs during gamete formation and development in most eukaryotes (reviewed by Burggren, 2015; Feng, Jacobsen, & Reik, 2010). In this section, we separately discuss the immediate (intragenerational effects) and longer term (inter‐ and transgenerational effects) of asexuality on epigenetic modifications and clarify when we discuss DNA‐dependent or autonomous epimutations.

#### 2.3.1 Immediate effects of asexual reproduction (within a generation)

Many of the immediate epigenetic effects of a transition to asexual reproduction in vertebrates likely stem from the lineage‐forming interspecific hybridization event. As noted in the preceding section on “the genomic consequences of asexuality,” novel parental genome interactions in hybrids may lead to DNA‐dependent changes in epigenetic marks, including a decrease in DNA methylation that can initiate transposable element derepression (e.g., O'Neill et al., 1998). Studies in plants suggest that other epigenetic markers may also vary after hybridization‐induced asexuality, such as histone acetylation and methylation (e.g., Jiao et al., 2018; Madlung & Wendel, 2013; Shi, Zhang, Ko, & Chen, 2015). However, changes in ploidy often accompany the examples of plant and animal hybridization that have been studied (e.g., Matos, Coelho, & Schartl, 2016; Shao et al., 2018). This co‐occurrence of hybridization and ploidy change makes it difficult to determine which of these major genomic changes (i.e., hybridization, polyploidization and asexual reproduction) leads to the observed epigenetic differences. To date, animal biologists have almost exclusively investigated the effects of interspecific hybridization on DNA methylation (e.g., Laporte et al., 2019; O'Neill et al., 1998; Xiao et al., 2013 & see Section 2.2.4 “Transposable element load”). Thus, a wider range of epigenetic marks such as histone modifications should be examined in vertebrates of similar ploidy to clarify the potential effects of hybridization, and hybridization‐induced asexuality on epigenetic variation.

A transition to asexuality may also increase the relative importance of phenotypic plasticity resulting from epigenetic variation as a mechanism leading to population persistence in a variable environment (Angers, Castonguay, & Massicotte, 2010; Castonguay & Angers, 2012; Verhoeven & Preite, 2014). Phenotypic plasticity, which can be defined as the ability of “individual genotypes to produce different phenotypes when exposed to different environmental conditions” (Pigliucci, Murren, & Schlichting, 2006), may often be regulated by epigenetic variation (e.g., Burggren & Crews, 2014; Ecker, Pancaldi, Valencia, Beck, & Paul, 2018; Hu & Barrett, 2017), and is predicted to be a key mechanism by which all organisms might cope with environmental change during a lifetime (reviewed by Snell‐Rood, Kobiela, Sikkink, & Shephard, 2018). Plasticity may be particularly important for clonal lineage persistence because all offspring of a single clonal lineage will be genetically identical; epigenetic modifications are the sole way they may differentially respond to environmental variation, in contrast to sexual siblings that also vary genetically (Castonguay & Angers, 2012; Verhoeven & Preite, 2014). Thus, in variable environments, epigenetically mediated plasticity may be critical for allowing clonal lineages to persist (Vogt, 2017).

To date, comparisons of epigenetic variation between sexual and asexual individuals, and the phenotypic consequences of these modifications, have mainly focused on DNA methylation in plants (reviewed by Verhoeven & Preite, 2014; Vogt, 2017). The potential for epigenetic modifications to contribute to phenotypic plasticity in asexual vertebrates has been best studied in diploid clonal lineages of the widespread *Chrosomus eos‐neogaeus* (Castonguay & Angers, 2012; Leung & Angers, 2018; Leung, Breton, & Angers, 2016, 2018; Massicotte & Angers, 2012; Massicotte, Whitelaw, & Angers, 2011). Data collected to date suggests that there is extensive randomly occurring and environment‐directed autonomous epigenetic variation within a *Chrosomus* clonal lineage (Massicotte & Angers, 2012; Massicotte et al., 2011). In particular, environment‐directed epimutations lead to most variation among predictable environments, while randomly occurring epimutations predominated in more variable environments (Leung et al., 2016). These data match theoretical predictions that environment‐directed epimutations contribute more to phenotypic plasticity and randomly occurring epimutations contribute more to diversifying bet‐hedging (Leung et al., 2016). To determine if epigenetic modifications make a larger contribution to beneficial plasticity in asexual versus sexual fish, we require further information about epimutation variation in clones compared to sexual parents and must test the effects of candidate epimutations on phenotypes and fitness.

#### 2.3.2 Longer term effects (over generations)

The genomes of most asexual clones do not undergo recombination among homologous chromosomes, so cannot purge deleterious mutations or combine new potentially beneficial genetic combinations (as reviewed in Section 2.2.3 “Deleterious mutation accumulation”). Thus, clonal lineages with higher beneficial phenotypic plasticity (general purpose genotype; Baker, 1965), may outcompete other lineages with lower beneficial plasticity in variable environments (Lynch, 1984). Epigenetic differences among clonal lineages are one potential mechanism generating this variation in plasticity. Thus, in variable environments, clonal selection may result in the dominance of lineages with higher epigenetic variation, which may be either DNA‐based or autonomous (Verhoeven & Preite, 2014). However, it is not yet clear if this clonal selection results in a higher level of epigenetic variation in successful asexual lineages compared to sexual congeners.

Changes in meiosis during gamete production in clones could also influence the stability of epigenetic markers if clones modify the normal resetting of epigenetic marks that occurs during this process (Verhoeven & Preite, 2014). Modes of asexual reproduction that bypass meiosis (e.g., mitotic parthenogenesis) should miss meiotic epigenomic resetting. However, all known clonal vertebrates undergo meiosis, albeit modified (Avise, 2008), so the prediction of increased epigenetic stability is not as applicable to vertebrate lineages as plants or invertebrates capable of mitotic egg production (Neiman et al., 2014; Verhoeven & Preite, 2014). However, as far as we are aware, the intergenerational and transgenerational stability of epigenetic modifications have not yet been compared between closely related sexual and asexual vertebrate populations. As well, while asexuality might not be an immediate effect on vertebrate epigenetic stability, asexual vertebrates can be good model species in which to study the stability of autonomous epigenetic variation due to their genetic homogeneity (e.g., Berbel‐Filho, Rodríguez‐Barreto, Berry, Garcia De Leaniz, & Consuegra, 2019; Fellous et al., 2018; Shao et al., 2018; and see Section 3.4 “Current research—What epigenetic mechanisms leading to variation in animal performance?”).

## 3 USING *FUNDULUS DIAPHANUS* X *F. HETEROCLITUS* CLONAL LINEAGES AS A MODEL SYSTEM IN WHICH TO STUDY THE GENOMICS OF ASEXUALITY AND EPIGENETIC EFFECTS ON PHENOTYPES

### 3.1 Asexual fish lineages as model organisms

Naturally occurring clonal fish, amphibians and reptiles should be good experimental subjects in which to study a number of questions about the genetic basis of reproductive incompatibilities (see Section 3.3 “Current research—What are the genomic causes and consequences of a transition to asexuality?”), because the formation of these asexual lineages prevents gene flow among parental species (Janko et al., 2018; Moritz et al., 1989). As well, understanding the genomic consequences of asexuality should help us better understand the potential costs and benefits of sex. The hybrid origin of vertebrate asexual lineages does make it difficult to disentangle the effect of asexuality and hybridization on genomic architecture, so comparisons with parental species, other closely related species, and sexual hybrids (if present) in a comparative framework will be critical for interpreting findings.

Clonal vertebrates can also help us better understand the regulation and effects of epigenetic variation (see Section 3.4 “Current research—What epigenetic mechanisms lead to variation in animal performance?”; Best et al., 2018; Laskowski et al., 2019; Vogt, 2017). This is because experiments testing the factors leading to variation of autonomous epigenetic modifications must control for background genetic variation, which can be done by using clone mates (reviewed by Best et al., 2018; Bossdorf, Richards, & Pigliucci, 2008; Hu & Barrett, 2017; Laskowski et al., 2019; Verhoeven & Preite, 2014; Vogt, 2017). For these studies, parthenogenic or gynogenetic animals are required, because sibling hybridogens will genetically differ due to the incorporation of meiotically produced sperm from sexual fathers (reviewed by Avise, 2008; Laskowski et al., 2019). Alternatively, hybridogens might be quite useful for studies testing for allele‐specific effects on DNA‐dependent epigenetic regulation in a common maternal genetic background (Laskowski et al., 2019).

**FIGURE 3:**
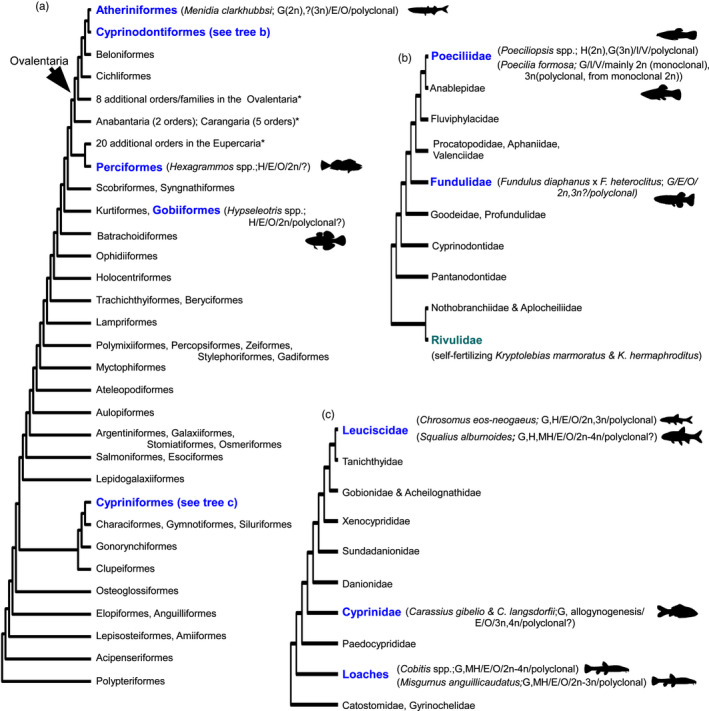
Ray‐finned fish (Actinopterygii) lineages with naturally occurring, asexual clonal lineages. (a) Simplified cladogram including major Actinopterygiian groups (based upon Hughes et al., 2018). Groups containing naturally occurring, asexual lineages are highlighted in blue and bolded. The two orders containing more than one asexual lineage, the (b) Cyprinodontiformes and (c) Cypriniformes are shown in greater detail. The Cyprinodontiform cladogram (b) is based upon Bragança, Amorim, and Costa (2018), which differs from the trees proposed by Pohl, Milvertz, Meyer, and Vences (2015) and Reznick, Furness, Meredith, and Springer (2017). The Cypriniformes cladogram (c) is based upon Stout, Tan, Lemmon, Lemmon, and Armbruster (2016), but alternative phylogenetic affinities have been proposed (e.g., Hirt, Arratia, Chen, & Mayden, 2017). The eight additional Ovalentaria orders denoted with the asterix (*) in (a) include Ambassidae, Mugiliformes, Pseudochromidae, Pomacentridae, Grammatidae, Opistognathidae, Gobiesociformes, and the Blenniiformes. The two Anabantaria and five Carangaria groups denoted with an asterix (*) in (a) include the Synbranchiformes and Anabantiformes (Anabantaria), and Centropomidae, Toxotidae, Carangiformes, Polynemidae, and Pleuronectiformes (Carangaria). The 20 additional Eupercaria groups noted with an asterix (*) in (a) are the Pempheriformes, Gerreiformes, Uranoscopiformes, Labriformes, Centrarchiformes, Moronidae, Ephippiformes, Lobotiformes, Lutjanidae, Haemulidae, Sciaenidae, Acanthuriformes, Pomacanthidae, Chaetodontiformes, Siganidae, Spariformes, Caproiformes, Priacanthiformes, Lophiiformes, Tetradontiformes. (a–c) The scientific names of known asexual lineages are written in italics, with reproductive mode(s) (Gynogenesis = G, Hybridogenesis = H, Meiotic hybridogenesis = MH), fertilization mode(s) (External = E, Internal = I), type(s) of development (Oviparous = O, Viviparous = V, and Ovoviviparous = Ov), ploidies (diploid = 2n, triploid = 3n,  tetraploid = 4n), and number of independent clonal lineage origins (monoclonal, polyclonal) noted in brackets, with a “?” if information is not known or remains equivocal (Avise, 2008; Collares‐Pereira et al., 2013; Cunha, Coelho, Carmona, & Doadrio, 2004; Cunha et al., 2011; Janko et al., 2012; Kimura‐Kawaguchi et al., 2014; Kuroda et al., 2018; Lamatsch & Stöck, 2009; Lampert, 2009; Morishima et al., 2008; Schmidt et al., 2011; Stöck et al., 2010; Suzuki et al., 2017; Warren et al., 2018). Note that parental leakage may occur in many of these lineages; see Lamatsch and Stock (2009) for further information. As well, additional genomic combinations and ploidies not included in this figure can be artificially produced (see Franek et al., 2019; Lamatsch & Stöck, 2009; Shao et al., 2018). The only known clade with self‐fertilizing fish (Rivulidae) is noted in green. Fish silhouettes for asexual groups are from PhyloPic (http://www.phylopic.org)

While all unisexual vertebrate lineages can be used to study the genomic causes and consequences of asexuality and the factors influencing epigenetic variation, ray‐finned fishes may be especially tractable experimental animals (Figure 3; reviewed by Best et al., 2018; Franěk et al., 2019; Laskowski et al., 2019; Vrijenhoek, 1994). This is because most asexual fishes are oviparious and have external fertilization, with the exception of asexual poecillids (Figure 3; Avise, 2008; Lamatsch & Stöck, 2009). So, when testing the factors leading to autonomous epigenetic change, it will be possible to more tightly control other, potentially confounding, early‐life environmental factors than in viviparous species with parental care (reviewed by Best et al., 2018; Laskowski et al., 2019; Verhoeven & Preite, 2014; Vogt, 2017). As well, the use of oviparous animals allows detection of transgenerational epigenetic effects a generation earlier than in viviparous animals. This is because pregnant, viviparous females (F0 generation), contain the F1 generation, which also contains the germ cells that contribute to the F2 generation, so these will be directly exposed to any epigenetic inducer. Therefore, true transgenerational effects cannot be detected until the F3 generation (reviewed by Best et al., 2018). However, in oviparous animals, transgenerational effects can be detected in F2's, facilitating studies of epigenetic stability (reviewed by Best et al., 2018).

All well‐characterized clonal fish are hybridogens or gynogens that require sperm from a parental species or another congener to activate embryonic development (Figure 3; Avise, 2008; Kimura‐Kawaguchi et al., 2014; Lamatsch & Stöck, 2009; Schmidt, Bond, Adams, & Hughes, 2011), further facilitating developmental studies that require precise control of fertilization timing (reviewed by Best et al., 2018; Laskowski et al., 2019; Verhoeven & Preite, 2014; Vogt, 2017). This sperm‐dependence means asexual fish normally live in sympatry with at least one parental sexual species, so ecologically relevant, common rearing conditions can be used when comparing sexual and asexual taxa (e.g., Kim, Waller, Aspbury, & Gabor, 2014). Perhaps most importantly, fish normally have higher fecundity and are often easier to care for in the laboratory than amphibians and nonavian reptiles (reviewed by Franěk et al., 2019).

Naturally occurring asexual species have been identified in five fish orders: the Cypriniformes, Gobiiformes, Atheriniformes, Cyprinodontiformes, and Perciformes (Figure 3; Avise, 2008; Kimura‐Kawaguchi et al., 2014; Lamatsch & Stöck, 2009; Schmidt et al., 2011). In addition, while not asexual, some populations of the self‐fertilizing hermaphrodite mangrove rivulus (*Kryptolebias marmoratus*; Harrington, 1961), and congener *Kryptolebias ocellatus* (Tatarenkov, Lima, Taylor, & Avise, 2009), are highly inbred, almost entirely homozygous, and thus essentially clonal. Therefore, the mangrove rivulus shares many of the benefits of asexual clonal lineages as models in which to examine the factors influencing autonomous epigenetic variation (Berbel‐Filho et al., 2019; Fellous et al., 2018). Mangrove rivulus also have some additional perks, because crosses can be made to conduct quantitative genetic studies and facilitate the mapping of genotype to phenotype (Kanamori et al., 2016; Kelley et al., 2016). It is quite possible there are still undiscovered asexual fish lineages, which may be identified by features such as a highly female skewed sex ratios or a high number of F1 hybrids without evidence of backcrossing (Avise, 2008; Beukeboom & Vrijenhoek, 1998). Furthermore, for experimental questions where natural clonal lineages are not required or tractable, there are many artificial fish asexual and isogenic lines currently available, and the potential for more to be created (e.g., Franěk et al., 2019; Shao et al., 2018; Spivakov et al., 2014).

In the following section, we review what is known about *Fundulus diaphanus* x *F. heteroclitus* asexual lineages, present some preliminary results from our laboratories, and discuss future research goals. We do not focus extensively on other asexual fishes, so we refer interested readers to reviews by Arai and Fujimoto (2013), Avise (2008, 2012, 2015), Lampert (2009), Lampert and Schartl (2008), Lamatsch and Stöck (2009), and Vrijenhoek (1994), as well as some of the recent research on asexual Poeciliidae (Alberici da Barbiano, Gompert, Aspbury, Gabor, & Nice, 2013; Gabor, Barbiano, & Aspbury, 2013; Schedina, Groth, Schlupp, & Tiedemann, 2018; Schlupp, Riesch, & Tobler, 2007; Stöck, Lampert, Möller, Schlupp, & Schartl, 2010; Warren et al., 2018), Hexagrammidae (e.g., Munehara, Horita, Kimura‐Kawaguchi, & Yamazaki, 2016; Suzuki, Miyake, Arai, & Munehara, 2019), Cyprinidae [*Carassius* (Gui & Zhou, 2010; Jiang et al., 2013; Li et al., 2018), *Chrosomus* (Lafond et al., 2019; Leung & Angers, 2018; Leung et al., 2016, 2018; Mee, 2014; Mee, Brauner, & Taylor, 2011; Mee & Taylor, 2012; Vergilino, Leung, & Angers, 2016), S*qualius alburnoides* (Collares‐Pereira & Coelho, 2010; Collares‐Pereira, Matos, Morgado‐Santos, & Coelho, 2013; Morgado‐Santos et al., 2017; Pereira, Ráb, & Collares‐Pereira, 2013)], *Misgurnus anguillicaudatus* (e.g., Kuroda et al., 2018; Kuroda, Fujimoto, Murakami, Yamaha, & Arai, 2019; Kwan, Ko, Jeon, Kim, & Won, 2019; Yamada et al., 2015) and *Cobitius* spp. (Choleva et al., 2012; Cunha, Doadrio, Abrantes, & Coelho, 2011; Janko et al., 2012, 2018; Ko, Yoon, Kim, & Park, 2015; Majtánová et al., 2016) for further information.

### 3.2 Asexual, clonal lineages of banded (*F. diaphanus*) and Common Killifish (*F. heteroclitus*)

The family Fundulidae is native to North and Central America and contains about 42 species of fish that inhabit a wide range of environmental conditions (Burnett et al., 2007; Eschmeyer's Catalog of Fishes, 2020; Scott & Crossman, 1998; Whitehead, 2010). The best studied of these species is the Common Killifish or mummichog (*Fundulus heteroclitus*), an extremely eury‐tolerant fish that has become a model system in comparative physiology, toxicology and evolutionary biology (Burnett et al., 2007; Crawford, Schulte, Whitehead, & Oleksiak, 2020; Lister, Van Der Kraak, Rutherford, & MacLatchy, 2011; Miller, Reid, Nacci, & Whitehead, 2019; Reid et al., 2017). In recent years, the genomics and ecophysiology of other species of *Fundulus* has also been studied in a comparative framework to better understand the mechanisms contributing to local adaptation to environmental stressors (Oziolor et al., 2019; Rodgers, Roach, Reid, Whitehead, & Duvernell, 2018; Whitehead, 2010). The popularity of Fundulid fishes is due to many factors, including the large variation in environmental tolerances seen across populations and species, their abundance and ease of collection from the wild, and relatively low‐cost and easy care in the laboratory (reviewed by Burnett et al., 2007; Dawley, 1992).

Hybridization occurs among many *Fundulus* species (e.g., Barbas & Gilg, 2018; Oziolor et al., 2019; Schaefer, Duvernell, & Campbell, 2016), but is only known to result in asexual hybrids when *F. diaphanus* and *F. heteroclitus* interbreed (Dawley, 1992). *F. diaphanus* and *heteroclitus* are nonsister species that last shared a common ancestor around 15–25 million years ago (Ghedotti & Davis, 2017). While *F. diaphanus* is normally found in freshwaters, and *F. heteroclitus* in brackish waters or marine environments (Fritz & Garside, 1974b), they do live in sympatry in a number of sites across North America and can form hybrids (Fritz & Garside, 1974a; Griffith, 1972; Hernández Chávez & Turgeon, 2007; Hubbs, Walker, & Johnson, 1943; Weed, 1921). It was not until Dawley (1992) conducted laboratory crosses to study allozyme heritability that F1 hybrid females were found to reproduce clonally, likely by gynogenesis. Diploid F1 clonal hybrids have been found in high numbers in two locations in Nova Scotia, Canada: the Saint Mary's River and Porter's Lake (also called Porters Lake), where they make up roughly 10% of the killifish community in the brackish water regions of these waterbodies (Dawley, 1992; Hernández Chávez & Turgeon, 2007; Mérette, Bourret, & Turgeon, 2009). Dawley (1992), Dawley, Chrzanowski, Phiel, Beaulieu, and Goddard (1999) and Mérette et al. (2009) also detected a small number (<1%) of triploid hybrids, suggesting that, perhaps similarly to *Chrosomus eos‐neogaeus* hybrids (Dawley et al., 1987), some unreduced clonal eggs may incorporate sperm.

Hernández Chávez and Turgeon (2007) found that there were multiple, different F1 hybrid *F. diaphanus* x *F. heteroclitus* clonal lineages in Porter's Lake and the Saint Mary's River estuary by genotyping hybrids with a combination of eight nuclear microsatellite loci and a restriction fragment length polymorphism in the D‐loop of the mitochondrial genome. The finding of at least four distinct clonal lineages in each site argues that asexual lineages have formed multiple times independently in both locations (Hernández Chávez & Turgeon, 2007; Mérette et al., 2009). We have recently compared the composition of clonal lineages present in Porter's lake today (2017–2018) to those present in 2004–2007 (Hernández Chávez & Turgeon, 2007; Mérette et al., 2009) by genotyping clones collected in 2017–2018 with the mitochondrial RFLP assay and five of the microsatellite loci used by Mérette et al. (2009) and comparing this data to representative clones from 2004–2007 (see Data S1 for further information). We found that the same major clonal lineage has remained dominant over the past decade (Clone A; Figure 5). We also found evidence of what may be subsequent mutations in this lineage (Data S1; Clones A.2 to A.5) and that some of the less common lineages have also persisted over the past decade in Porter's Lake (Figure 5, Clones B‐K). Finally, we found some potential new clonal lineages (Figure 5, Clones L‐P) not detected in 2004–2007 (Hernández Chávez & Turgeon, 2007; Mérette et al., 2009). However, we cannot exclude the possibility that new lineages were simply not detected in 2004–2007, as Hernández Chávez and Turgeon (2007) calculated that well over 200 individuals are needed per site to detect all clones, but only 134 were originally studied. Interestingly, three of the potential new clonal lineages contained alleles present in current *F. diaphanus* populations (Tirbhowan, 2019, see Data S1, Clones N‐P), but not normally found in the historical clonal lineages. Together, these data support the hypothesis that clonal lineage formation has occurred repeatedly and may still be occurring at a low rate. As well, the finding that the same major clone has remained dominant over the past decade leads to the hypothesis that Clone A is outcompeting other clones because it has a higher capacity for beneficial plasticity in response to environmental change; we hope to test this hypothesis by comparing clonal tolerance to environmentally relevant stressors. Importantly, further genomic analyses are in progress to unequivocally assign clonal lineages and study mutation accumulation (see Section 3.3 Current research—What are the genomic causes and consequences of a transition to asexuality?).

In both Porter's Lake and the Saint Mary's River in 2004, all hybrids had a *F. diaphanus* mitochondrial genome (Hernández Chávez & Turgeon, 2007). We genotyped fish collected from Porter's Lake in 2017–2018 and found that 86 out of 90 (95.6%) F1 clonal hybrids had a *F. diaphanus* mitochondrial genome. It is not clear if this bias in hybridization direction is due to prezygotic or postzygotic reproductive isolation. However, in vitro *F. heteroclitus* x *F. diaphanus* crosses could be reared to adulthood in the laboratory (Dawley et al., 1999; Fritz & Garside, 1974a), suggesting that prezygotic factors, such as mate choice, and not intrinsic genetic incompatibilities, are likely contributing to this bias in cross direction in the wild.

The majority of hybrids found in Porter's Lake and the Saint Mary's River sites also appear to be F1 clones, but there is evidence that a small proportion of sexual hybrids might exist (Dawley et al., 1999; Hernández Chávez & Turgeon, 2007; Mérette et al., 2009). In vitro crosses of Porter's Lake fish resulted in offspring of both sexes, and males survived to adulthood, but it is not yet known if these offspring are capable of sexual reproduction (Fritz & Garside, 1974a). Hernández Chávez and Turgeon (2007) also found that sympatric *F. diaphanus* and *F. heteroclitus* were more morphologically similar than allopatric populations, further suggesting that sexual hybrids exist and lead to introgression among species (Mérette et al., 2009). Indeed, in other locations in the Maritime provinces of Canada, sexually reproducing, but not asexual hybrids are found (Hernández Chávez & Turgeon, 2007). These data, in combination with Hernández Chávez and Turgeon (2007)'s finding that only a subset of *F. diaphanus* mitochondrial DNA haplotypes are found in clonal hybrids in Porter's lake, argue that only specific genetic combinations or environmental conditions result in clonally reproducing progeny (Hernández Chávez & Turgeon, 2007).

A common prediction for clonal lineages is that the reduction in population size and lack of recombination will lead to mutational meltdown, making them “evolutionary dead ends” (Lynch, Bürger, Butcher, & Gabriel, 1993). However, this process may take 10^4^–10^5^ generations to manifest (Lynch & Gabriel, 1990), and the high heterozygosity found in most hybrid clonal vertebrate genomes may buffer against the lack of new genetic diversity from meiotic recombination (Warren et al., 2018). The exact time of origin of the *F. diaphanus* x *F. heteroclitus* clonal lineages is not known, but they might have formed as recently as 70 years ago in Porter's Lake when this lake was first connected to the ocean, allowing the more freshwater tolerant *F. diaphanus* and brackish/salt water‐inhabiting *F. heteroclitus* to come into contact (Fritz & Garside, 1974b). Population genetic analysis of the mitochondrial genome also suggests that asexual hybrid origin is recent, as clonal haplotypes are at the edges of the mitochondrial network (Hernández Chávez & Turgeon, 2007). If these hybrids formed in their current location, they must be younger than ~12,000 years, as these regions of Nova Scotia were covered by the Wisconsinian glaciers until this point (Shaw et al., 2006). Further population genetic analysis is needed to definitively age clones, as has been done for other species [e.g., *Poeciliopsis*, (Quattro, Avise, & Vrijenhoek, 1992; Stöck et al., 2010)], but these populations are predicted to be too young to be experiencing mutational meltdown (Lynch & Gabriel, 1990). While measures of fitness have not been compared between parental species and the F1 clones, the F1 hybrids seem to have parasite loads (King, 2009) and salinity tolerances (Jonah, 2019) intermediate to that of their parent species, suggesting there is no major hybrid dysfunction in adult clonal females, beyond their asexuality. Nor does there appear to be any major heterosis or increased plasticity compared to parental species (Jonah, 2019), as would be predicted if the dominant clone was a true general‐purpose genotype (Baker, 1965).

As Dawley (1992) first highlighted, these clonal killifish lineages can be quite useful model organisms. This is because (a) they are gynogens, meaning they will have genetically identical offspring barring new mutation, unlike hybridogens [e.g., *Poeciliopsis* (Schultz, 1969), *Hexagrammos* (Suzuki, Arai, & Munehara, 2017), and *Hypseleotris* spp. (Schmidt et al., 2011)], (b) they are mainly diploid, facilitating genomic studies hoping to disentangle the effects of ploidy and hybridization‐induced asexuallity, unlike *Carassius* gynogenic triploid clones (Gui & Zhou, 2010) and many triploid gynogenetic *Cobitis* lineages (Majtánová et al., 2016), (c) they are externally fertilized, unlike the livebearing Poecillidae clones (*Poeciliopsis* and *Poecilia*), facilitating controlled laboratory breeding, and (d) their gynogenetic mode of inheritance appears to be more stable than some other clonal fish species [e.g., *Chrosomus* (Dawley et al., 1987), *Squalius alburnoides* (Pereira et al., 2013), *Misgurnusis* (Kuroda et al., 2019)]. Furthermore, there is a great deal of information about *Fundulus* spp. physiology (Burnett et al., 2007; Whitehead, 2010) and the genomic (Reid et al., 2017) and transcriptional variation (Whitehead & Crawford, 2005) associated with responses to a variety of environmental stressors (Brennan et al., 2018; Healy, Brennan, Whitehead, & Schulte, 2018), including salinity (e.g., Brennan, Galvez, & Whitehead, 2015; Kozak, Brennan, Berdan, Fuller, & Whitehead, 2014; Marshall et al., 2018; Scott, Rogers, Richards, Wood, & Schulte, 2004; Whitehead, Roach, Zhang, & Galvez, 2011; Whitehead, Zhang, Roach, & Galvez, 2013), temperature (e.g., Healy & Schulte, 2019; Whitehead & Crawford, 2006), hypoxia (e.g., Flight, Nacci, Champlin, Whitehead, & Rand, 2011), and pollutants (e.g., Reid et al., 2016; Whitehead et al., 2012). Coupled with strong genomic resources for *Fundulus* spp*.* (Johnson et al., 2020; Miller et al., 2019; Reid et al., 2017), killifish clonal lineages should be a useful system in which to study genomic and epigenomic evolution.

### 3.3 Current research—What are the genomic causes and consequences of a transition to asexuality?

According to the “balance hypothesis,” asexual reproduction can evolve when species have accumulated enough divergence to disrupt meiosis, yet not enough to compromise viability of their hybrids (Janko et al., 2018; Moritz et al., 1989). Therefore, we are working to identify the proximate mechanisms leading to asexual lineage formation in *Fundulus* hybrids. Specifically, we are interested in understanding how sequence divergence and global genome architecture may interact to destabilize meiosis in *Fundulus* hybrids. Disentangling the consequences of asexual reproduction in vertebrate hybrids is a tricky endeavor. The *Fundulus* system, with the likely presence of both sexually and asexually reproducing hybrids, allows us to use a comparative framework; this may be an extremely powerful way to distinguish between the consequences of hybridization alone and the combination of hybridization and asexual reproduction.

#### 3.3.1 How does genome architecture differ between *F. heteroclitus* and *F. diaphanus*?

Genome architecture can be defined as “the totality of non‐random arrangements of functional elements in the genome” (Koonin, 2009), in which we include the karyotype (chromosome number, arrangement and centromere position), the amount and distribution of repetitive elements (e.g., transposable elements and satellite repeats), as well as gene synteny. Genome architecture directly influences many processes of interest to evolutionary biologists, such as recombination rate patterns, adaptive introgression and speciation (Lynch, 2007). As genome architecture is directly linked to chromosome alignment during meiosis, it is critical that we gain a better sense of genome organization in *Fundulus* to understand hybridization outcomes and the transition to asexuality.

**FIGURE 4:**
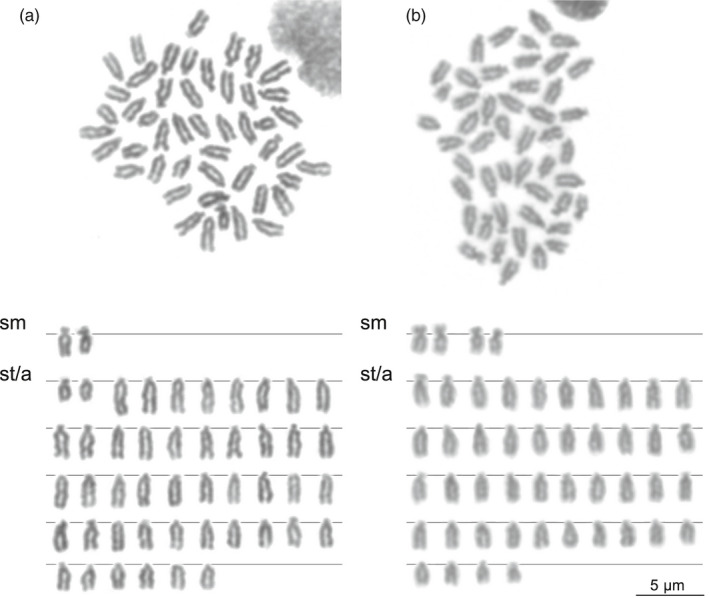
Karyotype of *Fundulus* fishes from Porters Lake, NS, Canada. (a) Giemsa‐stained karyotype of a male *Fundulus heteroclitus* individual (2N = 48, NF = 50). (b) Giemsa‐stained karyotype of a male *Fundulus diaphanus* individual (2N = 48, NF = 52). The karyotypes are highly similar to previously published data in other populations of *Fundulus* (Chen, 1971). a, acrocentric; sm, sub‐metacentric; st, sub‐telocentric

Fundulidae have rather conserved karyotypes (typically 2N = 46–48, NF = 48–52; Arcement & Rachlin, 1976; Chen, 1970, Chen, 1971), yet *F. diaphanus* has a genome size that is ~10% larger than *F. heteroclitus* (Dawley, 1992). This suggests a higher repetitive element load in *F. diaphanus*, which could result from reduced selection efficiency against selfish genetic elements (assuming *F. heteroclitus* has a larger effective population size, being a more widely distributed marine species, Reid et al., 2017). We will perform a comparative study of the genomes of *F. heteroclitus* and *F. diaphanus*, with a particular emphasis on repetitive elements, which are known to correlate well with genome size in eukaryotes (Dufresne & Jeffery, 2011; Lynch & Conery, 2003). Our initial observations are consistent with previously published karyotypes for *F. heteroclitus* and *F. diaphanus males* from other populations (Figure 4; Chen, 1971). We are now pursuing a deeper comparative karyotype characterization using finer cytogenetic markers such as chromomycin A_3_ staining (GC‐rich heterochromatin), C‐banding (constitutive heterochromatin), as well as fluorescent in situ hybridization (using probes targeting ribosomal genes and telomeres). These techniques may reveal subtle, yet stronger global genome architecture divergence than what can be seen with Giemsa‐based karyotypes, as observed previously in other fish systems (Dion‐Côté et al., 2017; Symonová et al., 2013). It will also be interesting to contrast these results with that seen in other species of Fundulidae, such as *F. grandis*, which also hybridize with *F. heteroclitus*, but produce fertile, sexually reproducing F1 offspring (Barbas & Gilg, 2018).

#### 3.3.2 Do sexually reproducing hybrids exist and do specific genotype combinations result in asexual reproduction?

Long thought to be a maladaptive process, hybridization is now recognized as a frequent source of beneficial genetic novelty in animals (Abbott et al., 2013; Harrison & Larson, 2014; Runemark, Vallejo‐Marin, & Meier, 2019). Thus, sexually reproducing *Fundulus* hybrids may provide a conduit for new adaptive alleles to enter the gene pools of the parental species (e.g., Oziolor et al., 2019). For example, higher freshwater tolerance in Northern versus Southern populations of *F. heteroclitus* (Scott et al., 2004), is hypothesized to be the result of adaptive introgression of “freshwater tolerance alleles” from the freshwater‐preferring *F. diaphanus* (Hernández Chávez & Turgeon, 2007). A potentially confounding factor for the classification of hybrids from Porter's Lake might be the action of mutational processes and gene conversion at microsatellite loci, which would either increase or decrease heterozygosity, respectively. Genomic data should help to exclude such technical issues, clarify the presence of sexually reproducing hybrids in Porter's Lake, and determine if sexual hybrids act as an introgression vehicle for the transfer of novel alleles into either of the parental species (e.g., Edelman et al., 2019). If the presence of sexually reproducing hybrids is confirmed, the *Fundulus* spp. system could also be an excellent comparative system to study the genetic factors that result in the production of sexual versus asexual hybrid offspring.

As noted in Section 3.2 “Asexual, clonal lineages of Banded (*F. diaphanus*) and Common Killifish (*F. heteroclitus*),” the majority of asexually reproducing hybrids from Porter's Lake carry a *F. diaphanus* mitochondrial haplotype [95.6% in 2017–2018 (Data S1) and 100% in 2004 (Hernández Chávez & Turgeon, 2007)]. In addition, some highly introgressed *F. heteroclitus* individuals have an *F. diaphanus* mitochondrial haplotype, which further supports the hypothesis that there are sexually reproducing hybrids (Hernández Chávez & Turgeon, 2007). Is this asymmetry in breeding direction related to cytonuclear incompatibilities between the *F. heteroclitus* mitochondrial DNA haplotype and the *F. diaphanus* nuclear background, or is this the result of female mating preference in the wild? Considering that sexually reproducing hybrids collected at other locations (e.g., NS, PEI; Hernández Chávez & Turgeon, 2007) carry a *F. heteroclitus* mitochondrial haplotype, it seems likely that this asymmetry is a consequence of some ecological factors (e.g., female mate choice) and not due to major cytonuclear incompatibilities. We are currently carrying out controlled laboratory crosses to investigate female mate choice, male‐male competition, and salinity tolerance in conspecific and reciprocal hetero‐specific crosses to determine the reasons for this bias in hybridization direction.

#### 3.3.3 How is meiosis modified in *Fundulus* asexual hybrids?

**FIGURE 5:**
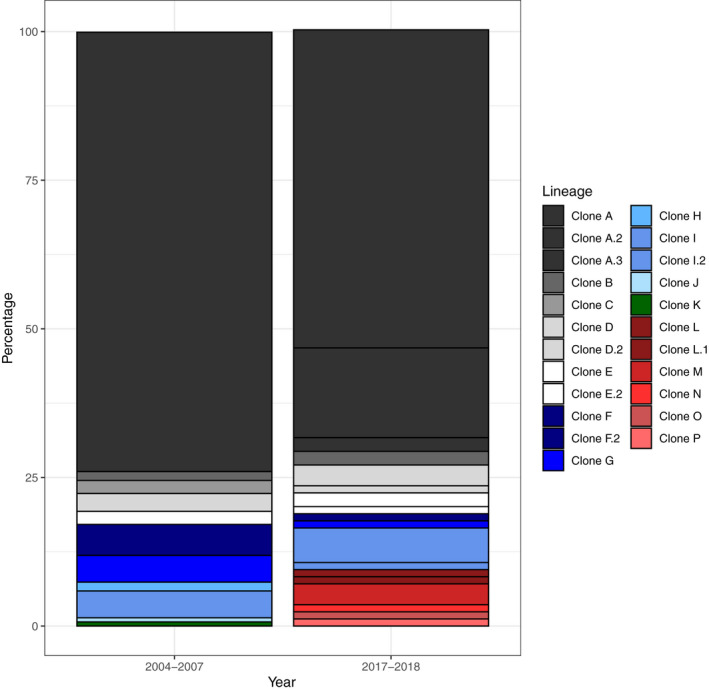
Clonal lineages of *F. diaphanus* x *F. heteroclitus* hybrids present in Porter's Lake (Nova Scotia, Canada) in 2004–2007 and 2017–2018. 2017–2018 Clonal lineages were identified with a combination of five microsatellite loci (FhCA‐1, FhCA‐21, Fhe57, FhATG‐B103, and Fhe113) and a mitochondrial D‐loop restriction fragment length polymorphism assay following the methods of Hernández Chávez & Turgeon, (2007) and Mérette et al., (2009), with some modifications described in the Appendix S1 section “clonal genotyping.” Representative individuals from some clonal lineages found in 2004–2007 (Clones A, B, C, F, G, H, and I) were analyzed at the same time as the 2017–2018 fish (*n* = 86 assigned to clonal lineages) to calibrate allele sizes between laboratories. Data from 2004–2007 is reproduced from Hernández Chávez and Turgeon (2007) and Mérette et al., (2009) (*n* = 134 asexual diploids from clonal lineages A to K). The same main clonal lineage (Clone A) predominated in Porter's Lake in 2004–2007 and 2017–2018. We also found evidence for subsequent mutations in Clone A (Clone A.2 and A.3) and other historical lineages (e.g., Clone D.2). Clonal lineages not previously detected by Mérette et al. (2009), were also found (Clones L to P; colored in shades of red); this includes three lineages (N, O, P) with alleles at FhCA‐1 and Fhe57 found in current day *F. diaphanus* populations (Tirbhowan, 2019), but not other clonal lineages, suggesting these lineages are recently formed. As in 2004 (100%; 29 of 29 clones), the majority of hybrids in Porter's Lake in 2017–2018 (95.6%; 86 of 90 clones) had a *F. diaphanus* mother, as indicated by their mitochondrial DNA haplotype (Data S1)

Asexually reproducing vertebrates produce their eggs through some modified version of meiosis (Kuroda et al., 2018, 2019; Nabais, Pereira, Cuñado, & Collares‐Pereira, 2012; Nabais, Rampin, & Collares‐Pereira, 2013; Neaves & Baumann, 2011). Based on observations in other asexual fish lineages, earlier genetic studies (Hernández Chávez & Turgeon, 2007; Mérette et al., 2009), and our most recent clonal lineage assessment (Figure 5), asexual *Fundulus* hybrids most likely produce their eggs by premeiotic endoduplication, thus preserving ploidy and heterozygosity. However, this still needs to be formally tested, as other modes of egg production, while unlikely, are possible (e.g., endoduplication during meiosis, oogonial fusion and mitosis; e.g., Neaves and Bauman, 2011; Newton et al., 2016). We aim to use a combination of cytogenetic approaches to investigate the mode of egg production in asexual hybrids compared to parental species. Specifically, we will use histological sections of ovaries combined with semi‐quantitative DAPI staining to determine the DNA content of germ cell throughout egg formation (Newton et al., 2016). This should allow us to determine at which stage the genome is duplicated, therefore maintaining ploidy in these clonal lineages. In addition, we will develop immunofluorescence techniques that, in combination with cytogenetic approaches, should allow us to determine whether sister chromosomes pair and recombine (Kuroda et al., 2018; Pereira et al., 2013), thus maintaining heterozygosity in asexual *Fundulus* lineages (Hernández Chávez & Turgeon, 2007).

#### 3.3.4 Do asexual lineages have higher rates of genome rearrangements and repetitive DNA accumulation?

As discussed in Section 2.2 “The genomic consequences of asexuality,” meiosis modification releases constraints on global genome architecture as homologous chromosomes are no longer required to align during meiosis I ( Flot et al., 2013; Fradin et al., 2017). In addition, transposable elements, especially if mobilized as a result of hybridization, may serve as substrate for recombination between nonhomologous loci, thus resulting in chromosome rearrangements (Deininger, Moran, Batzer, & Kazazian, 2003). Asexual lineages may also lose male‐specific genes or genes involved in meiosis that are no longer needed (Yin et al., 2018, but see Warren et al., 2018), or display high rates of transposable element excision (Bast et al., 2019) that would result in genome size reduction over time (this is especially true in fishes that contain high loads of “cut‐and‐paste” DNA transposons; Symonová & Suh, 2019).

We will investigate whether *Fundulus* hybrids have genome rearrangements compared to parental species by combining cytogenetic approaches with single‐molecule sequencing technologies. While cytogenetics is a powerful and affordable strategy to investigate large‐scale genome rearrangements across many individuals, single‐molecule sequencing may reveal smaller scale changes (e.g., small inversions or translocations of a few kilobases that would go undetected by cytogenetics; Weissensteiner & Suh, 2019). Therefore, this integrated “cytogenomic” approach will allow documenting small‐scale (within chromosome) and large‐scale rearrangements (among chromosomes) to provide a more detailed picture of potential genome rearrangements in asexually reproducing *Fundulus*. Furthermore, if sexually reproducing *Fundulus* hybrids are present in the system, we can directly test if asexual *Fundulus* hybrids have higher rates of genome rearrangements than sexually reproducing hybrids.

Hybridization and meiotic recombination disruption are predicted to lead to the accumulation of transposable elements (but see Section 2.2.4 “Transposable element load”). We might expect hybrids to have a larger genome, but, in nematodes, reproductive mode shifts have been associated with genome shrinkage, probably due to the loss of male‐specific genes (Yin et al., 2018). Combined with cytometry, sequencing data will allow us to test these predictions in asexually reproducing *Fundulus*. The comparison between asexually and sexually reproducing hybrids will be of the upmost importance to disentangle the consequences of hybridization and asexual reproduction.

#### 3.3.5 Do asexual lineages have higher rates of deleterious mutation accumulation?

Asexual organisms are predicted to accumulate deleterious mutations due to the lack of recombination (Hill & Robertson, 1966), reduced effective population size (Balloux et al., 2003; Orive, 1993) and Muller's ratchet (Felsenstein, 1974; Muller, 1964), a phenomena that has been confirmed in asexually reproducing *Daphnia*, freshwater snails and stick insects (Bast et al., 2018; Neiman, Hehman, Miller, Logsdon, & Taylor, 2010; Paland & Lynch, 2006). A particularity of the clonal *Fundulus* system is that these are likely evolutionary “young” clonal lineages (potentially <70 years old, see Section 3.2 “Asexual, clonal lineages of Banded (*F. diaphanus*) and Common Killifish (*F. heteroclitus*)”; Fritz & Garside, 1974a). While this means *Fundulus* spp. clones in Porter's Lake might not have had enough time to accumulate deleterious mutations, it potentially allows us to witness the very first deleterious mutations that accumulate, even before fixation. We will test for nonsynonymous, frameshift and nonsense mutation accumulation in RNA‐sequencing and whole‐genome sequencing datasets generated to answer other questions. Preferential expression of one parental allele over the other may also represent a mechanism to mitigate deleterious mutation expression (see following section).

#### 3.3.6 How is the transcriptome remodeled in asexually reproducing hybrids?

Gene expression differences may lead to significant phenotype changes even between closely related species (Pavey, Collin, Nosil, & Rogers, 2010). In hybrids, the interaction of two divergent genomes can lead to novel patterns of gene expression that can generate novel phenotypes (e.g., “hybrid vigor”; Landry, Hartl, & Ranz, 2007) and even underlie reproductive isolation (Mack & Nachman, 2017). Recent work investigating gene expression changes across five asexual stick insects species has revealed repeated convergent changes of genes involved in meiosis in the reproductive tract (Parker et al., 2019a). More surprisingly, repeated masculinization (increased expression of male‐biased genes) was also observed in these stick insect species (Parker et al., 2019b). We will ask whether and how transcriptional networks are remodeled in asexual *Fundulus* hybrids. We are particularly interested in testing for parental allele bias among expressed genes as a mechanism to mitigate deleterious allele accumulation, which can be tested by comparing wild asexual individuals to controlled crosses. In addition, it will be exciting to look at gene expression profiles in ovaries as well as in tissues that may regulate important responses to environmental stressors, such as the gills, which must respond to changes in salinity as a primary site of osmoregulation. Finally, these transcriptomic datasets will allow us to test directly for transposable element derepression in these asexual hybrids.

### 3.4 Current research—What epigenetic mechanisms lead to variation in animal performance?

A major aim of comparative physiology is to determine the mechanisms that contribute to differences in animal performance within and among individuals, populations and species in the wild (Mykles, Ghalambor, Stillman, & Tomanek, 2010). One mechanism that we still have much to learn about is the potential contributions of autonomous epigenetic variation to differences in animal function and fitness (reviewed by Burggren, 2015; Burggren & Crews, 2014; Hu & Barrett, 2017; Verhoeven et al., 2016). In particular, we need to learn more about: (a) how specific environmental factors influence the epigenome, (b) how the resulting epigenetic variation influences phenotype, performance and fitness and, (c) the stability of epigenetic inheritance (Hu & Barrett, 2017; Skvortsova et al., 2018; Verhoeven et al., 2016). In this section, we discuss how studies in clonal killifish hybrids might contribute to these research questions.

#### 3.4.1 How does the environment influence the epigenome?

To learn more about how the environment may influence autonomous epimutations, it is critical to control for DNA‐dependent epigenetic effects (Bossdorf et al., 2008; Laskowski et al., 2019). Indeed, inbred lines of vertebrates have been the model of choice for medical physiologists testing the links between autonomous epigenetic modifications and disease (Cavalli & Heard, 2019). However, ecological physiologists interested in animal performance and fitness in the wild may prefer to study animals that are “the products of natural selection” (Laskowski et al., 2019; Vogt, 2017). Recent work in such natural clonal population of fishes does suggest that there is a clear signature of autonomous environment‐mediated DNA methylation (Berbel‐Filho et al., 2019; Leung et al., 2016; Massicotte et al., 2011). However, how other types of epigenetic marks might vary, and the phenotypic effects of this epigenetic variation, are largely unknown for natural animal populations (Burggren, 2015; Cavalli & Heard, 2019; Duncan et al., 2014; Hu & Barrett, 2017; Verhoeven et al., 2016).

We hope to use *F. diaphanus* x *F. heteroclitus* clonal lineages to test how exposure to ecologically relevant environmental variation, such as changes in water salinity, affects epigenetic modifications in fish after acclimation. In particular, we hope to disentangle the effects of environment‐directed (those that consistently vary in response to a given environmental condition) and nontargeted autonomous epigenetic marks in fishes (e.g., Berbel‐Filho et al., 2019). Of course, these clones are hybrids with novel gene combinations compared to parental species. So, to determine the generality of potential results linking environmental exposures to specific epimutations, it will be necessary to also test the epigenetic responses of genetically variable parental species.

#### 3.4.2 How does epigenetic variation influence phenotype and performance?

Over the past decades, studies in genetic model organisms have shown that epigenetic modifications can lead to changes in gene expression that may translate to differences in organismal phenotypes (Feil & Fraga, 2012). However, there is still much to learn about the linkages between specific epigenetic marks and phenotype, even in genetic model organisms (Cavalli & Heard, 2019; Duncan et al., 2014). Furthermore, studies in natural populations have mainly focused upon genome wide changes in DNA methylation (Hu & Barrett, 2017; Verhoeven et al., 2016), and have rarely studied epigenetic variation known to be autonomous (but see Berbel‐Filho et al., 2019; Fellous et al., 2018; Thorson et al., 2017). Thus, we cannot yet accurately predict the effects of specific autonomous epimutations on phenotype in natural populations, and know much less about the phenotypic effects of other types of epimutation beyond DNA methylation in wild animal populations (Hu & Barrett, 2017).

Linking specific epimutations to phenotype will be difficult, as many epigenetic changes will occur in concert, so establishing causation will require more than correlational studies. Even in clonal lineages which have no differences in DNA‐dependent epigenetic modifications, the plethora of autonomous epigenetic modification will be hard to tease apart. One option will be to conduct epigenetic editing of candidate sites and then test for effects on gene regulation and candidate phenotypes (Josipović et al., 2019; Liu et al., 2016). Furthermore, as we learn more about how specific epigenetic marks might influence gene expression and phenotypes in natural vertebrate populations, a later goal should be to study combined profiles of different types of epigenetic marks (e.g., DNA methylation, specific forms of histone modifications, and small RNAs) that may interact to influence phenotype (Burggren, 2015). In terms of the *Fundulus* asexual lineages, a particularly interesting question is if epigenetic factors (DNA‐dependent or autonomous) contribute to the differences in the production of sexual versus asexual hybrid offspring in different populations (Hernández Chávez & Turgeon, 2007).

#### 3.4.3 How stable are epigenetic marks?

To determine the evolutionary role of autonomous (non‐DNA‐dependent) epigenetic modifications in vertebrates, we must first gain a better understanding of the persistence of epigenetic marks (Lind & Spagopoulou, 2018; Skvortsova et al., 2018; Verhoeven et al., 2016). In the past decade, it has become clear that certain epigenetic modifications produced as a result of environmental factors in plants and some invertebrates can persist, and continue to modify gene expression and physiology long after the initial stressor has ended (reviewed by Hollick, 2017; Quadrana & Colot, 2016; Serobyan & Sommer, 2017; Skvortsova et al., 2018). Furthermore, these modifications can contribute to local adaptation (Schmid et al., 2018). However, the extent to which epigenetic modifications are transmitted across generations in vertebrates, which undergo more substantial germline epigenetic reprogramming, is less clear (Heard & Martienssen, 2014; Hu & Barrett, 2017; Lim & Brunet, 2013; Perez & Lehner, 2019; Radford, 2018; Skvortsova et al., 2018). Recent studies suggest that the answer to this question may be taxa specific: in mammals efficient epigenetic reprogramming of most histone and DNA methylation occurs in the early embryo and both the male and female germline (Skvortsova et al., 2018; Wang et al., 2014), while in early zebrafish embryos the paternal DNA methylome are stably inherited at least intergenerationally (Murphy, Wu, James, Wike, & Cairns, 2018; Ortega‐Recalde, Day, Gemmell, & Hore, 2019; Skvortsova et al., 2019; Zhu, Xu, Wang, & Liu, 2019), but many histone modifications are not (Ortega‐Recalde et al., 2019; Skvortsova et al., 2019; Zhu et al., 2019). Surprisingly, there appear to be differences in epigenetic reprogramming among species within a group of vertebrates, such as the teleost fishes; DNA methylation is extensively reprogrammed during development in medaka, unlike in zebrafish (Wang & Bhandari, 2019; Zhu et al., 2019). These data suggest that the potential for transgenerational epigenetic inheritance should be assessed in a wider range of vertebrate taxa to fully understand the evolutionary potential of this mode of information transfer.

The study of clonal lineages might help to clarify the extent of intergenerational and transgenerational autonomous epigenetic inheritance (reviewed by Laskowski et al., 2019; Verhoeven and Preite, 2014; Vogt, 2017). For example, Fellous et al. (2018) examined global changes in methylation in an isogenic line of self‐fertilizing mangrove rivulus (*Kryptolebias marmoratus*) and found that reprogramming occurs at a later stage than in zebrafish, is more extensive and extends longer into development. However, we do not yet know how stable specific modifications are in asexual lineages. Because the paternal genome contribution to transgenerational epigenetic inheritance are quite important in fish (Murphy et al., 2018; Ortega‐Recalde et al., 2019; Skvortsova et al., 2019; Zhu et al., 2019), studying gynogenetic lineages is one way to test for potential maternal contributions to transgenerational epigenetic inheritance. The potential presence of both sexual and asexual hybrids between *F. diaphanus* x *F. heteroclitus* may make this a particularly interesting comparative system. Of course, asexual vertebrate lineages, with modified meiosis, may vary from the typical developmental program in sexual lineages, so we may not be able to generalize epigenetic reprogramming mechanisms (Verhoeven & Preite, 2014). Whatever the case, information about the presence, and extent of, transgenerational epigenetic inheritance in asexuals compared to sexuals will allow evolutionary biologists to test the hypothesis that epigenetic modifications play an important role in facilitating asexual hybrid lineage persistence (Castonguay & Angers, 2012; Leung et al., 2016; Massicotte et al., 2011; Verhoeven and Preite, 2014; Vogt, 2017). Comparing the extent of autonomous and DNA‐dependent transgenerational epigenetic inheritance among more (e.g., Clone A) and less dominant killifish clonal lineages in Porter's Lake (e.g., Clones B‐L) may also clarify the contributions of each type of epigenetic variation in clonal lineage success.

## 4 SUMMARY

The field of comparative physiology has been strongly influenced by Krogh's Principle, that “for a …number of problems there will be some animal of choice, or a few such animals, on which it can be most conveniently studied” (Krogh, 1929). As comparative physiologist work to understand the importance and mechanisms of epigenetic regulation on phenotypic variability (reviewed by Burggren & Crews, 2014), the ability to use genetically identical clones may be an ideal “Kroghian model” to control for the potentially interacting effects of genetic variation while maintaining ecological relevance (Laskowski et al., 2019). We hope that studying clonal *Fundulus* spp. that derive from Common Killifish, a well‐established model species in comparative animal physiology (reviewed by Burnett et al., 2007; Crawford et al., 2020), will provide us with an opportunity to merge a deep knowledge of physiological mechanisms with potential underlying epigenetic variation.

Furthermore, fish of the family Fundulidae have become a key model system in which to study the genetic basis for adaptation (e.g., Brennan et al., 2018; Crawford et al., 2020; Oziolor et al., 2019) and speciation (e.g., Barbas & Gilg, 2018; Fuller, McGhee, Schrader, 2007) in natural populations. By combining genomic and cytogenetic data from *F. diaphanus* x *F. heteroclitus* clones with currently available genomic resources for the Fundulidae clade (e.g., Johnson et al., 2020; Reid et al., 2017), we hope to better understand the mechanisms by which genomes are regulated and evolve in response to hybridization and asexual reproduction.

## CONFLICT OF INTEREST

None declared.

Box 1 The Bernatchez Lab circa 2015. (a) PhD defense celebration for Dr. Anne‐Marie Dion‐Côté, with (b) Lake Whitefish (*Coregonus clupeaformis*) cake designed by Dr. Laura Benestan. (c) Lab méchoui party at Louis' house in Summer 2015.

Anne—I was a postdoctoral fellow in the Bernatchez laboratory from 2013‐2016; this was a very exciting time, as there were at least eight postdoctoral fellows, fifteen graduate students and three technicians working with Louis! This group included diverse array of biologists conducting research in population genetics, evolutionary biology and molecular ecology (Louis' core research themes), along with some physiology, molecular biology, genomics, and conservation biology. Furthermore, these scientists were interested in questions in both pure and applied research and came from a range of nationalities and first languages. While I had not left Canada (my home country), my time in the Bernatchez Lab and Quebec City felt like a truly international experience. I was able to experience the bumps of learning a new language and culture in an intellectually fertile, supportive and fun working environment. The ability to form teams of interdisciplinary researchers and provide them with the tools and freedom to develop new ways of thinking is one of Louis' many skills. I will forever be amazed at how he was able to track progress on the dozens of projects in his lab, keep up with editorial responsibilities, and organize parties—he is a force to be reckoned with! The working relationships and friendships developed during my time in the Bernatchez laboratory have shaped my own laboratory's research, and I am extremely grateful that he took this wayward comparative physiologist into his fold.

Anne‐Marie—I was a research assistant in the Bernatchez laboratory from 2009‐2010, and then a PhD student from 2011‐2015. I came from an MSc in molecular and cell biology studying the biochemistry of DNA repair, and worked in Louis' laboratory for almost two years with the ultimate goal of learning about molecular ecology and evolution. Those were exciting times: next‐generation sequencing was becoming a technological pillar in molecular ecology (Louis was always an early adopter of new technologies!) and many talented and motivated students and postdocs from diverse cultures were joining the laboratory. I will forever be in debt to Louis for giving me the opportunity to learn about (and ultimately choose!) a whole new field in which I had no training. For my PhD, I leveraged my background in molecular biology to tackle overlooked (if not forgotten!) questions in speciation, with Louis' entire trust. During my PhD, I travelled to international conferences and had the opportunity to build international collaborations in Czech Republic and Sweden which are still ongoing to this day. Louis' laboratory is a genuine scientific family with no borders—*merci pour tout!*

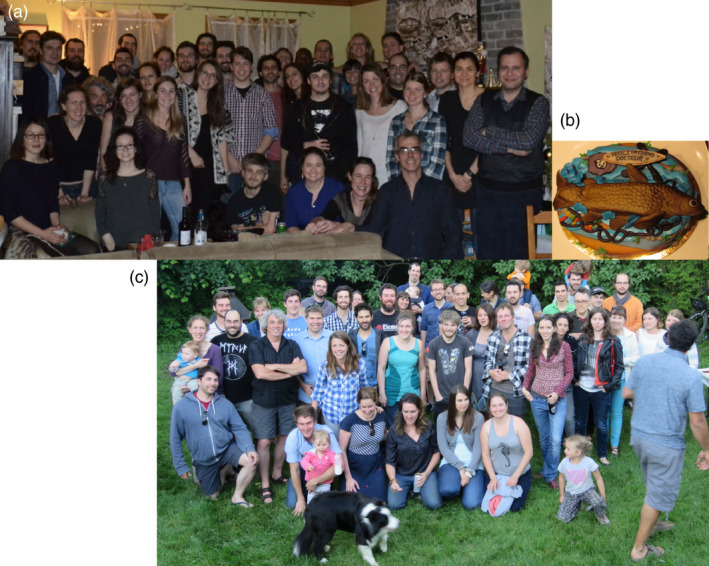

## Supporting information

Supplementary Material

## Data Availability

The microsatellite genotypes from this study are provided as Appendix S1.
