# Supplementary material for: Using asexual vertebrates to study genome evolution and animal physiology: Banded (Fundulus diaphanus) x Common Killifish (F. heteroclitus) hybrid lineages as a model system
Source: Evol Appl. 2020 May 4;13(6):1214–39. doi: 10.1111/eva.12975 (PMC7359844; doi:10.1111/eva.12975)
Supplement: Supplementary file 1 — Supplementary Material [file EVA-13-1214-s001.zip › eva12975-sup-0001-Supinfo.docx]

**Supplemental information - Methods**

*Clonal genotyping*

We tested if the same clonal lineages detected in 2004-2007 [(Hernández Chávez and Turgeon 2007; Mérette et al. 2009)](https://paperpile.com/c/fbD1ME/E7lN+3K8H) were found in 2017-2018 in Porter’s Lake, Nova Scotia, Canada (44.7433°N, 63.2972°W). To do this, we obtained tissues from Mérette et al. (2009) for Clones A, B, C, F, G, H collected in 2007 and also collected *F. diaphanus* x *F. heteroclitus* asexual hybrids from Porter’s lake from May-October of 2017 and 2018. In 2017-2018, fish were collected with minnow traps, dip netting, and seine nets at sites within the original 2004-2007 sampling area described by Mérette et al. (2009), following methods approved by the Saint Mary’s University Animal Care Committee protocols under a Department of Fisheries and Oceans Canada permit (Jonah, 2019). During collections, we used a morphological measurement protocol described by Mérette (2009) to tentatively identify fish as *F. diaphanus*, *F. heteroclitus* or asexual hybrids. Fish were then brought back to Saint Mary’s University and fin clips were collected for DNA extractions to confirm species identity via genotyping.

We extracted DNA with commercially available DNA extraction kit (either Sigma or Omega-Biotek) and quantified DNA by measuring absorbance at 260 nm (Spectromax M3 M series Microplate reader, Molecular devices). We then amplified four species-specific nuclear microsatellite loci following the general methods described by Hernández Chávez and Turgeon (2007; *Fh*CA-1, *Fh*CA-21 and *Fhe*57), with Mérette et al. (2009)’s decreased annealing temperature and increased primer concentrations, from 2007 and 2017-2018 samples. We assessed allele sizes on an Applied Biosystems 3500xL Genetic Analyser by running samples with the GeneScanTM 600 LizTM dye Size Standard v2.0 (Applied Biosystems) and analysing data with GeneMarker® version 2.7.4. In addition, a 441 bp region of the mitochondrial D-loop was amplified to distinguish the *F. diaphanus* from *F. heteroclitus* mitochondrial genomes with an *Hph*I restriction fragment length polymorphism (RFLP) assay. *Hph*I cuts twice in this region of the D-loop in *F. diaphanus* and only once in *F. heteroclitus* (Hernández Chávez and Turgeon 2007). We ran out the RFLP assay on a 4% agarose gel stained with ethidium bromide to visualize bands. Due to poor D-loop amplification in some *F. heteroclitus* samples, we designed new *F. diaphanus* and *F. heteroclitus* consensus primers to amplify a 600 bp region of the *Fundulus* D-loop (Forward primer 5’- TTAACCCCCACCCCTAGCTC -3’, Reverse primer 5’- GCACTGTGAAATGTCAACTGAA -3’) and used these primers to re-amplify any problem samples. This new D-loop RFLP assay resulted in bands at 211, 166 and 215 bp in *F. diaphanus* and 211 and 381 bps in *F. heteroclitus* (Tirbhowan, 2018).

We identified 2017-2018 fish as hybrids if they had one *F. diaphanus* allele and one *F. heteroclitus* allele at at least two of the three species-specific microsatellite loci (*Fh*CA-1, *Fh*CA-21 and *Fhe*57; see Supplemental data). To identify which clonal lineage each fish belonged to, we then amplified the two additional nuclear microsatellite loci (*Fh*ATG-B103 and Fhe113) used by Hernández Chávez and Turgeon (2007) and Mérette et al. (2009) to distinguish clonal lineages. We found allelic dropout at FhATG-B103 when amplifying the 2007 control samples using the PCR conditions of Mérette et al. (2009). Therefore, we reduced our annealing temperatures from 55°C to 50°C, under which conditions we were able to replicate the findings of Mérette et al. (2009) at FhATG-B103. We also found evidence for non-specific binding at Fhe113, but were able to recover the alleles studied by Mérette et al. (2009). We note any non-specific binding in our data (Supplemental data). We did find a shift of approximately 4-5 bp in the allele sizes when measured on the Applied Biosystems 3500xL Genetic Analyser at Saint Mary’s University compared to the results from Mérette et al. (2009), so our allele sizes (Supplemental Information) are slightly smaller than those found by Mérette et al. (2009). By combining allelic information from these five microsatellite loci and the mitochondrial genome, we assigned individual fish to the clonal lineages first detected in 2004-2007 (A to I; Mérette et al. 2009) or to a new lineage J to N. In situations where hybrids had a matching genotype at all loci except for one nuclear allele, potentially suggesting a new mutation, we assigned them as a subset of a major clonal genotype (e.g. Clone A.2). We did the same when all nuclear alleles were identical, but a different mitochondrial genome was found to be conservative in our clone numbers, even though this does most likely indicate a unique hybridization event.

*Chromosome suspension and imaging*

Chromosome suspensions were prepared from spleen and kidney tissue. Briefly, individuals were injected with 0.1% colchicine (BioShop Canada) at a dose of 1 mL/100 g of body weight and left to rest for 45 min. Individuals were then sacrificed with 200 mg/L benzocaine (BioShop Canada) and the anterior kidney and spleen were sampled promptly. A single cell suspension was then prepared by scraping the sampled tissue against a screen for CD-1^TM^ size 40 mesh (Millipore Sigma) with 1-2ml of hypotonizing solution (KCl 0.075M). The cell suspension was then transferred to a 15 mL tube and incubated in 5 mL of 0.075M KCl for 15 min at room temperature. Cells were then pre-fixed with a few drops of freshly-prepared, ice cold fixative (3 methanol:1CH_3_COOH). Cells were then centrifuged at 250xG and fixed in 5 mL of ice cold fresh fixative for 20 min. This was followed by three washes with 5 mL of ice cold fresh fixative, and the cells were finally resuspended in 1 mL of fixative before being spread onto microscope slides (VWR Superfrost ® Plus Micro slides). Slides were dehydrated (ethanol 70%, 80%, 95%, 100% ice-cold, 3 min each) and then stained for 20 min in Giemsa (Thermo Fisher). Metaphase plates were imaged on a Leica DMRB 301-371.010 microscope at 1000X.
